# Anæmia

**Published:** 1881-11

**Authors:** 


### ANÆMIA

[From Dr. Hall's Health at Home, or Family Doctor Book.]

IS literally "without blood," and means that the blood is poor, thin, watery; hence the pale face and wan appearance ; there is no animation, no life. This condition is brought on by frequent bleedings from any part of the body, severe sickness, long-protracted disease, bad food, insufficient eating. It is often found in young girls ; even the lips are pale sometimes, also the tongue. There is a general debility, feeble pulse, nervousness, and palpitation of the heart, attended generally with cold feet, costiveness, bad taste in the mouth of mornings, and very irregular appetite, with changing pains to any and all portions of the body. Such persons take cold very easily, are subject to chills from slight causes, have no stamina, no vitality, with the result very generally that they die early or soon fall into a consumptive condition.

In all such cases prompt means should be taken to build up the general health, and these should be persevered in for weeks and many months, if necessary, for, to give up because there seems to be but little change, because the progress toward health seems slow, is to abandon the person to a premature death.

The first thing to be done is to regulate the eating to thrice a day; nothing whatever between meals. For supper take a cup or two of hot drink, with some cold bread and butter, or bread crust broken into tea.

Any drink at meals should be warm, with cold bread and butter and a piece of fresh meat of any kind for breakfast, and nothing else. Dinner same, adding one vegetable and nothing else; no dessert unless ripe, raw fruit or berries or grapes.

Great pains should be taken to keep the feet warm. If one plan does not do, after a systematic, persevering effort, try another, remembering that until the feet become warm, and remain so habitually, there is no real approach toward health. Cold feet show that the blood does not properly circulate through the system, and when that is the case there is no health, there is always disease, which can be removed in one way only—vigorous and healthful circulation of the blood. To that end take a dose of liver pills once a month, and between times use castor oil or salts. But there is a far better way—walk or work in the open air during the forenoon, and also during the afternoon, beginning with one hour at a time, increasing ten minutes every day, until an amount of time is spent in this way which will secure not only a regular action of the bowels, but will get up a good appetite and a vigorous digestion. This plan will seldom fail if persevered in; if it fails all others will fail. The point is to ride on horseback, or walk, or work in the open air enough every day to get really hungry. If spirits or liquor are taken in these frequent forms of disease, the result is to quicken the pulse and thus wear out the strength more rapidly, without adding any ingredient to the blood to increase it in quality or quantity. Ale and beer and porter sometimes seem to increase the flesh, but never by any means add to the real strength of the body, much less to improve the blood. Various preparations of iron are given in these bloodless, pale-faced conditions of the system, but there is only one safe, healthful, certain and natural way of adding iron to the blood, and that is to supply it through a vigorous digestion of substantial meat and bread. These will give all the iron which the blood requires. Warm feet, regular bowels, a good appetite—these are all important. They are essentials; they will cure if cure is possible, if several hours are given to exertive exercise in the open air every day, rain or shine.

